# Polymeric nanoparticle‐based delivery of TRAIL DNA for cancer‐specific killing

**DOI:** 10.1002/btm2.10019

**Published:** 2016-08-19

**Authors:** Stephany Y. Tzeng, David R. Wilson, Sarah K. Hansen, Alfredo Quiñones‐Hinojosa, Jordan J. Green

**Affiliations:** ^1^ Dept. of Biomedical Engineering, Johns Hopkins University School of Medicine Baltimore MD; ^2^ The Institute for Nanobiotechnology and the Translational Tissue Engineering Center Johns Hopkins University School of Medicine Baltimore MD; ^3^ Dept. of Neurosurgery Johns Hopkins University School of Medicine Baltimore MD; ^4^ Dept. of Oncology Johns Hopkins University School of Medicine Baltimore MD; ^5^ Dept. of Ophthalmology Johns Hopkins University School of Medicine Baltimore MD; ^6^ Dept. of Material Science and Engineering Johns Hopkins University Baltimore MD

**Keywords:** nanoparticles, non‐viral gene therapy, poly(beta‐amino ester), polymer, TRAIL

## Abstract

Lack of specificity in cancer therapeutics severely limits the efficacy of many existing treatment modalities. The use of Tumor Necrosis Factor‐related Apoptosis‐Inducing Ligand (TRAIL) is of interest to the field due to this protein's ability to cause cell death specifically in cancer cells without harming the surrounding healthy tissue. Here, we report that polymeric nanoparticles, based on synthetic poly(beta‐amino ester)s (PBAEs) and containing DNA, are able to selectively transfect cancer cells in vitro over healthy cells of the same tissue type. Moreover, PBAE‐based nanoparticles containing TRAIL DNA are able to transfect several human cancer cell cultures in vitro and cause cell death. While certain cell types, including human glioblastoma (GBM), showed resistance to TRAIL, we found that the expression of TRAIL‐binding surface proteins was predictive of each cell type's resistance to TRAIL therapy. We demonstrate a non‐viral nanomedicine approach to cancer gene therapy that can improve cancer specificity via both biomaterial selection and through the use of cancer‐targeting genetic cargo.

## 1 Introduction

The ability to deliver DNA is attractive for clinical applications, including cancer therapy. Because aberrant gene expression plays a major role in diseases like cancer,^1^, ^2^ the use of nucleic acids themselves as the therapeutic agent presents a method to address the root cause of the disease, potentially curing or ameliorating diseases.^3^, ^4^, ^5^, ^6^, ^7^, ^8^ Despite its promise, the translation of gene therapy has been slowed by the difficulties of delivering DNA and other nucleic acids, which are large, hydrophilic, charged molecules that must penetrate the cell membrane and subsequent intracellular barriers for efficacy. While viruses tend to be highly effective delivery vehicles for gene transfer, concerns with individual vectors regarding excessive immune response, high rate of mutagenesis, and limitations to the size of the cargo that they can carry have limited their translation to the clinic.^9^, ^10^

The field of nanomedicine has recently produced many non‐viral gene delivery agents based on biomaterials that are capable of facilitating intracellular delivery of DNA and can be engineered for high transfection efficacy in relevant cells while minimizing toxicity.^11^, ^12^, ^13^ Poly(β‐amino ester)s (PBAEs), a class of synthetic, cationic polymers, have been found to be effective as non‐viral gene delivery agents. They are easy to synthesize, effective at binding to DNA, and hydrolytically degradable under physiological conditions, which decreases their cytotoxicity. PBAEs have been shown in our prior work to be successful in transfecting cancer cells both in vitro^14^, ^15^, ^16^, ^17^ and in vivo.^18^, ^19^, ^20^, ^21^ Moreover, PBAEs have also been shown to have cell‐type specificity based on their chemical structures,^22^, ^23^ with many PBAE‐based nanoparticles showing innate specificity in transfecting cancer cells over healthy cells from the same tissue type, independent of cell division rate.^17^, ^19^

In addition to biomaterial‐mediated specificity for cancer cells, gene therapy can utilize downstream transcriptional targeting and specialized protein activity to target and kill cancer cells, such as through the exogenous expression of a cytotoxic protein.^24^ We sought to evaluate cancer cell survival in vitro in response to polymeric delivery of the apoptotic gene tumor necrosis factor (TNF)‐related apoptosis‐inducing ligand (TRAIL), which binds to death receptors on the cell surface to trigger cell death. Because TRAIL‐binding death receptors are generally overexpressed in cancer cells compared to healthy cells, and because PBAE‐based nanoparticles demonstrate biomaterial‐mediated cancer specificity, we hypothesized that the combination would lead to enhanced cancer‐specific cell death.^25^ In this study, we engineered PBAE/DNA nanoparticles for gene delivery to several cancer cell types and examined their specificity for transfection of cancer cells over healthy cells derived from non‐cancerous tissue of the same type. We also investigated to what extent the non‐viral delivery of DNA encoding the TRAIL gene could cause cell death in various cancer cell lines. Finally, we examined mechanisms of resistance in cancer cells lines that were less responsive to TRAIL treatment.

## 2 Materials and methods

### 2.1 Polymer synthesis

**Figure 1:**
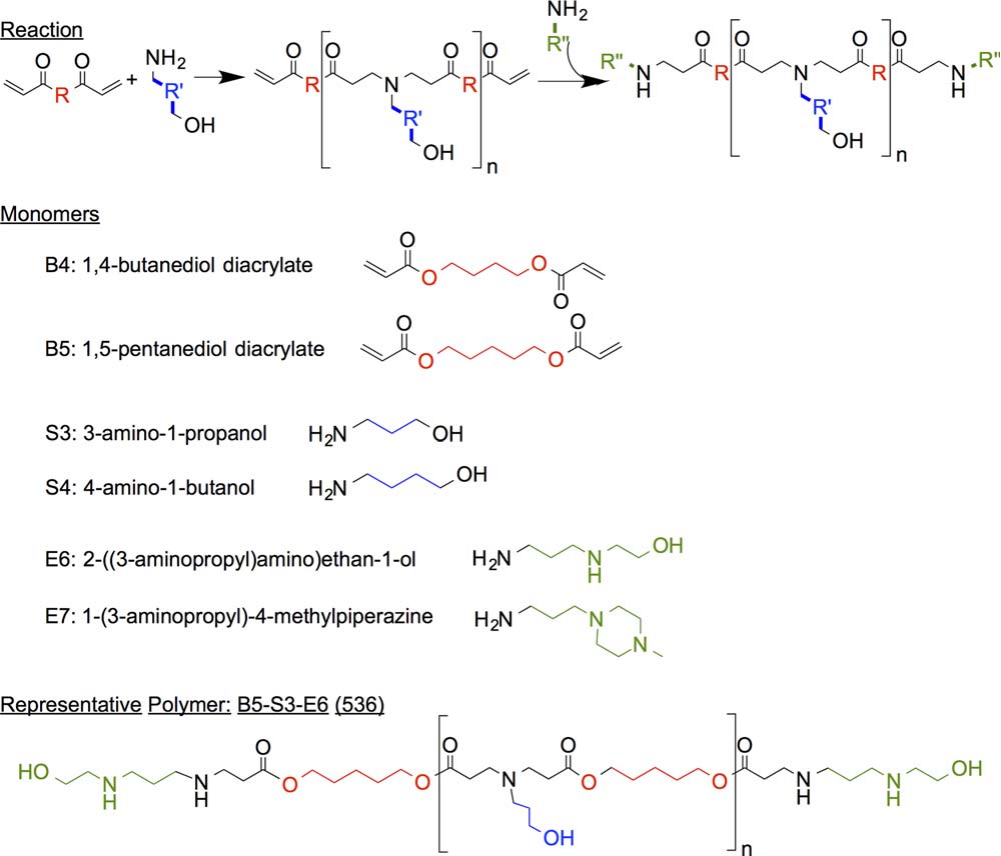
PBAEs were synthesized from small molecule monomers using Michael addition reactions to create linear, alternating copolymer, endcapped molecules

Poly(beta‐amino ester)s (PBAEs) were synthesized as previously described^17^ using the small molecules in Figure 1. Briefly, one diacrylate‐terminated backbone monomer (B) was polymerized with one primary amine‐containing sidechain monomer (S) in a neat solution by stirring for 24 hr at 90°C, forming the base polymer via Michael addition. This base polymer was dissolved in anhydrous tetrahydrofuran (THF) and mixed with one end‐cap small molecule (E), then stirred at room temperature for 1 hr. The end‐capped PBAE was then precipitated into diethyl ether, washed twice, and left under vacuum for 48 hr for complete removal of ether. The dry PBAE was dissolved in anhydrous DMSO at 100 mg/ml and stored at −20°C in small aliquots to minimize freeze‐thaw cycles. Polymers used in the study described in this report were chosen from top candidates found in previous work.

### 2.2 Nanoparticle‐mediated gene delivery

For optimization of nanoparticle formulations and transfection protocols for each cell line, a plasmid coding for enhanced green fluorescence protein (pEGFP‐N1, purchased from Elim Biopharmaceuticals, Hayward, CA, abbreviated “GFP,” and amplified by Aldevron, Fargo, ND) was used as a marker of successful transfection. Cancer cells used in this study, their type, their source, and their culture conditions are listed in Table 1; healthy cells used are listed in Table 2. Cells were seeded into 96‐well plates 1 day before transfection at a density of 15,000 cells/well in 100 μl complete culture medium. For cells grown in serum‐free conditions (JHGBM‐276, ‐612, and ‐965 cells), 96‐well plates were coated with 20 μg/ml mouse laminin (Sigma‐Aldrich, St. Louis, MO) before seeding cells. Cells were incubated at 37°C overnight for attachment.

**Table 1:** Cancer cell types, sources, and culture conditions

Name (abbr. name)	Cancer type	Species	Source	Complete culture medium
H446	Small‐cell lung cancer	Human	Dr. Christine Hann, Department of Oncology, Johns Hopkins University	RPMI + 10% FBS, 2 mM l‐glutamine, 1% pen/strep, 1 mM sodium pyruvate, 10 mM HEPES, 1.5 g/L NaHCO_3_
MDA‐MB‐231 (MDA)	Triple‐negative metastatic breast cancer	Human	ATCC (American Type Cell Culture, Manassas, VA)	High‐glucose DMEM + 10% FBS, 1% pen/strep
BxPC‐3	Pancreatic cancer	Human	Dr. Zeshaan Rasheed, Department of Oncology, Johns Hopkins University	High‐glucose DMEM + 10% FBS, 1% pen/strep
MeWo	Melanoma (metastatic to lymph node)	Human	Dr. Martin Pomper, Department of Radiology, Johns Hopkins University	High‐glucose DMEM with pyruvate and l‐glutamine + 10% FBS, 1% anti‐anti
MCA‐RH7777	Hepatocellular carcinoma (HCC)	Buffalo rat	ATCC	High‐glucose DMEM with pyruvate and l‐glutamine + 10% FBS, 1% pen/strep
U87 MG (U87)	Glioblastoma (GBM)	Human	Dr. Michael Lim, Department of Neurosurgery, Johns Hopkins University	High‐glucose DMEM with pyruvate and l‐glutamine + 10% FBS, 1% pen/strep
JHGBM‐276	Glioblastoma (GBM) Brain Tumor Initiating Cell (BTIC) primary culture	Human	Dr. Alfredo Quiñones‐Hinojosa, Department of Neurosurgery, Johns Hopkins University	DMEM/F12 (1:1) + B‐27 serum‐free supplement, 1% anti‐anti, 20 ng/ml bFGF, 20 ng/ml epidermal growth factor (EGF)
JHGBM‐319	Glioblastoma (GBM) primary culture	Human	Dr. Alfredo Quiñones‐Hinojosa, Department of Neurosurgery, Johns Hopkins University	DMEM/F12 (1:1) + 10% FBS, 1% anti‐anti
JHGBM‐612	Glioblastoma (GBM) Brain Tumor Initiating Cell (BTIC) primary culture	Human	Dr. Alfredo Quiñones‐Hinojosa, Department of Neurosurgery, Johns Hopkins University	DMEM/F12 (1:1) + B‐27 serum‐free supplement, 1% anti‐anti, 20 ng/ml bFGF, 20 ng/ml epidermal growth factor (EGF)
JHGBM‐965	Glioblastoma (GBM) Brain Tumor Initiating Cell (BTIC) primary culture	Human	Dr. Alfredo Quiñones‐Hinojosa, Department of Neurosurgery, Johns Hopkins University	DMEM/F12 (1:1) + B‐27 serum‐free supplement, 1% anti‐anti, 20 ng/ml bFGF, 20 ng/ml epidermal growth factor (EGF)

**Table 2:** Non‐cancer/healthy cell types, sources, and culture conditions

Name (abbr. name)	Tissue type	Species	Source	Complete culture medium
F34	Fetal neural progenitor cell (fNPC) primary culture	Human	Dr. Alfredo Quiñones‐Hinojosa, Department of Neurosurgery, Johns Hopkins University	DMEM/F12 (2:1) + B‐27 serum‐free supplement, 1% anti‐anti, 20 ng/ml bFGF, 20 ng/ml epidermal growth factor (EGF), 10 μg/ml leukemia inhibitory factor (LIF), 50 mg/ml heparin
BRL‐3A	Liver (hepatocytes)	Buffalo rat	ATCC	MEM+Glutamax + 10% FBS, 1% pen/strep, 1x non‐essential amino acids (NEAA), 2 mM l‐glutamine, 1 mM sodium pyruvate
hTERT‐HPNE	Pancreas	Human	Dr. Zeshaan Rasheed, Department of Oncology, Johns Hopkins University	Low‐glucose DMEM/M3BaseA (3:1) + 5% FBS, 250 μg/ml dextrose, 10 μg/ml EGF

To form PBAE/DNA nanoparticles, GFP DNA was diluted in 25 mM sodium acetate buffer (NaAc, pH 5) to 0.06 mg/ml. PBAEs were diluted in 25 mM NaAc and added to the diluted DNA solution in a 1:1 (vol/vol) ratio, resulting in 25:1 to 90:1 mass ratio of PBAE:DNA (w/w). The PBAE/DNA mixture was incubated at room temperature for 10 min for nanoparticle self‐assembly, then added to the cells in 96‐well plates. The final ratio of nanoparticle suspension‐to‐culture media was 1:5 (vol/vol). 600 ng DNA (5 μg/ml) and 15–54 μg PBAE (125–450 μg/ml) were added per well. Cells were incubated with nanoparticles for 4 hr for all cell types except MDA and JHGBM‐276, ‐319, ‐612, and ‐965 cells, which were incubated with particles for 2 hr. All nanoparticles and media were then replaced with 100 μl/well of fresh, complete culture medium. Non‐specific toxicity of the nanoparticles was measured after 24 hr using MTS assay (Cell Titer 96^®^ Aqueous ONE, Promega, Madison, WI). Transfection efficacy was observed using a fluorescent microscope (Axiovert Observer A.1, Zeiss) and using flow cytometry after 48 hr, using an Accuri C6 flow cytometer (BD Biosciences, Franklin Lakes, NJ) with Hypercyt high‐throughput sampler and reader (Intellicyt Corp., Albuquerque, NM).

For comparisons between healthy and cancerous cells from the same tissue type, the culture medium was changed immediately before addition of nanoparticles in order to ensure that there would be no differences in media conditions that could affect transfection efficacy or toxicity. For brain (JHGBM‐276 and fNPC) and liver (MCA‐RH7777 and BRL‐3A) cells, the normal culture media for non‐cancerous cells were used for transfection media. For pancreas cells (BxPC‐3 and hTERT‐HPNE), the pancreatic cancer cell medium was used in order to maintain 10% serum in the transfection medium, for better comparison to most of the other cell lines. Leading nanoparticle formulations (Table 3) were chosen for use in later studies with a functional plasmid.

**Table 3:** Leading nanoparticle formulations for cancer cell transfection

Cell line name	Cell type	Polymer name, polymer:DNA mass ratio (w/w)	GFP transfection efficacy (%)	Non‐specific toxicity (%)	TRAIL‐mediated killing (%)	Data source
H446	Lung cancer	447, 75 w/w	32 ± 2	10 ± 7	68 ± 2	Supporting Information Figure 1
BxPC‐3	Pancreatic cancer	447, 50 w/w	33 ± 1	26 ± 3	64 ± 3	Supporting Information Figure 2
MeWo	Melanoma	447, 50 w/w	80.6 ± 0.3	0 ± 2	13 ± 5	Supporting Information Figure 3
MDA‐MB‐231	Breast cancer	447, 60 w/w	56 ± 5	44.5 ± 0.6	43 ± 3	Reference^15^
U87	Glioblastoma	446, 60 w/w	51 ± 4	24 ± 2	2 ± 3	Supporting Information Figure 4
JHGBM‐276	Glioblastoma (primary culture)	537, 25 w/w	53 ± 4	2 ± 2	−4 ± 5	Reference^19^
JHGBM‐319	Glioblastoma (primary culture)	447, 25 w/w	62 ± 1	28 ± 2	27 ± 4	Reference^17^, ^19^
JHGBM‐612	Glioblastoma (primary culture)	447, 25 w/w	39 ± 3	16 ± 4	21 ± 2	Reference^19^
JHGBM‐965	Glioblastoma (primary culture)	537, 25 w/w	40 ± 4	69 ± 1	31 ± 1	Reference^19^

### 2.3 Nanoparticle characterization: nanoparticle size and zeta potential

PBAE nanoparticle hydrodynamic diameter was determined by nanoparticle tracking analysis (NTA) using a Nanosight NS500 with a 532 nm laser at 25°C (Nanosight, Amesbury, UK). Three independent samples were prepared successively for each nanoparticle formulation at a DNA concentration of 0.005 µg/µl by mixing equal volumes of DNA and polymer solutions in 25 mM NaAc. After 10 min to allow for nanoparticle formation, each sample was diluted in 150 mM PBS (pH = 7.4) to give a particle concentration of 20–80 particles per frame with dilution ratios of 1:100, 1:200, or 1:400. Three 60‐s captures were used to assess particle size for each sample with solution advancement between captures.

Zeta potentials of PBAE nanoparticles were measured by electrophoretic light scattering in disposable zeta cuvettes at 25°C using a Malvern Zetasizer NanoZS (Malvern Instruments, Marlvern, UK) with a detection angle of 173° and analyzed with the Smoluchowsky model. Three samples of each nanoparticle formulation were prepared successively in the same manner as for NTA but were instead diluted 1:4 in 150 mM PBS to a total volume of 800 µl.

To assess size and morphology by electron microscopy, nanoparticles at 60 w/w ratios for polymers 447 and 537 were prepared in 25 mM sodium acetate at a DNA concentration of 0.005 mg/ml. Six microliters of the respective sample was then loaded onto a corona plasma treated carbon film 400 square mesh TEM grid and allowed to dry over 1 hr, after which the grids were quickly dipped in water and allowed to fully dry. TEM images were captured using a Philips CM120 (Philips Research, Briarcliffs Manor, New York).

### 2.4 Nanoparticle characterization: particle formation and polymer‐DNA binding

Gel electrophoresis experiments were performed using 1% agarose gels with 1 µg/ml ethidium bromide run at 100 V for 25 min. Nanoparticles were formed at their respective w/w ratios by mixing equal volumes of DNA and polymer in 25 mM sodium acetate 10 min before loading the gel. Each sample was supplemented with a loading buffer of 30% glycerol immediately before loading. Gels were visualized under UV using a Gel Logic 200 Imaging System (Kodak).

### 2.5 Nanoparticle‐mediated gene delivery of Tumor Necrosis Factor‐related apoptosis‐inducing ligand (TRAIL)

Cells were seeded into 96‐well plates as described above. Using the leading PBAE/DNA formulations for each of the human‐derived cell types in Table 1 found from the initial experiments with GFP, nanoparticles were formed with DNA coding for GFP‐TRAIL fusion protein (pEGFP‐TRAIL, Addgene plasmid 10953,^26^ abbreviated “GFP‐TRAIL”) and used to transfect cells using the protocol described above. For each formulation tested, nanoparticles formed with GFP DNA were also made and used as a control. Forty‐eight hours after transfection, cell viability was measured using an MTS assay to quantify metabolic activity. TRAIL‐mediated killing was assessed qualitatively by microscopy and was also calculated by normalizing the metabolic activity of GFP‐TRAIL‐transfected cells to that of GFP‐transfected cells using the same nanoparticle formulation. Recombinant human TRAIL protein (rhTRAIL, Life Technologies) was added to the media of some cells in the absence of nanoparticles as a control.

### 2.6 Measurement of TRAIL and TRAIL receptor expression via polymerase chain reaction (PCR) and Western blot

To elucidate the reasons for lack of response to GFP‐TRAIL transfection by some cell types, PCR was used to verify that the GFP‐TRAIL gene used was in fact being transcribed. U87 glioblastoma cells were used as an example of a cell type that showed high expression after transfection with GFP but little or no response to transfection with GFP‐TRAIL. U87 cells were seeded into a 12‐well plate at 1.5 × 10^5^ cells/ml with 1 ml per well. With *n* = 3 replicates, cells were transfected with either GFP or GFP‐TRAIL using the top nanoparticle formulation found for U87 cells (listed in Table 3). After 48 hr, cells were lysed and harvested using TRIzol (Life Technologies) for RNA isolation and RT‐PCR analysis. Glyceraldehyde 3‐phosphate dehydrogenase (GAPDH) was analyzed as the housekeeping gene for comparison between samples.

Western blot and PCR were also used to measure different cell lines' expression levels of four surface proteins with TRAIL‐binding capacity: death receptors DR‐4 and DR‐5 and “decoy” receptors DcR‐1 and DcR‐2. Cells were collected from culture flasks using Accutase (Life Technologies) to minimize cleavage of surface proteins and seeded into 12‐well plates for PCR or 6‐well plates for Western blot. After 72 hr, cells were harvested, using TRIzol for PCR and cell scrapers on ice and in the presence of PBS with protease and phosphatase inhibitors. Cell lysates were stored at −80°C until use.

### 2.7 Statistics

Values displayed on graphs are shown as mean ± *SEM* of three wells for cell experiments or mean ± SD of three independently prepared particle aliquots for particle characterization. Graphpad Prism 6.0 was used for all statistical tests. *t* tests were used to compare transfection between pairs of cancer cells and healthy cells from the same tissue type. For polymer optimization screens for each cell type, a one‐way ANOVA was used to show statistically significant differences in transfection and viability from the commercially available reagent Lipofectamine 2000 tested at the same dosage. Statistical significance was designated as follows: ****p < .0001; ***p < .001; **p < .01; *p < .05.

## 3 Results

### 3.1 Nanoparticle optimization and characterization

An array of PBAE/DNA nanoparticle formulations with varied polymer structure (Figure 1) and dosing were evaluated for efficacy in different cancer cell types. The polymer naming convention “Bx‐Sy‐Ez,” or “xyz” for short, refers to “x” carbons between acrylate groups in the constituent “B” backbone monomer, “y” carbons between the amine and alcohol groups in the constituent “S” sidechain monomer, and “z” as a specific constituent amine containing “E” end‐capping group. For example, PBAE polymer B4‐S4‐E7 or “447” is poly(1,4‐butanediol diacrylate‐co‐4‐amino‐1‐butanol) endcapped with 1‐(3‐aminopropyl)‐4‐methylpiperazine. Polymers tested were primarily chosen based on their success in transfecting cancer cells in previous work.^15^, ^17^, ^19^ PBAE polymers 447, 446, and 537 at weight‐weight ratios to DNA between 25 and 75 were the optimal formulations as listed in Table 3. These nanoparticle formulations were chosen for further studies in each cell type based on maximal GFP expression ranging from 32 to 81% and minimal non‐specific toxicity ranging from 2 to 45%. Full graphs of the transfection efficacy and safety of the full range of polymers tested are shown in Supporting Information Figures 1–4 (Supporting Information Figure S1 shows the PBAE results in H446 lung cancer cells, Supporting Information Figure S2 in BxPC‐3 pancreatic cancer cells, Supporting Information Figure S3 in MeWo melanoma cells, and Supporting Information Figure S4 in U87 glioblastoma cells) and PBAE transfection optimization in additional cell types can be found in the literature.^15^, ^17^ Formulations that were considered for use in further studies were ones that caused the highest transfection while causing < 30% non‐specific toxicity. The PBAE formulations used in TRAIL‐mediated killing studies were chosen for each cell type based on the initial screening and optimizational.

### 3.2 Nanoparticle characterization

**Figure 2:**
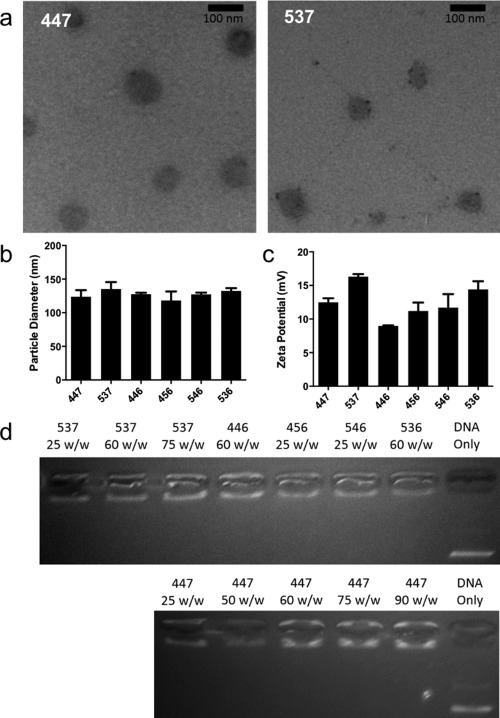
(a) TEM images of the two top PBAE/DNA nanoparticle formulations showed a mean size of approximately 100 nm. All nanoparticle formulations had a (b) mean hydrodynamic diameter between 100 and 150 nm determined via NTA and (c) mean zeta potential between positive 9–16 mV. (d) All nanoparticle formulations were demonstrated to fully retard DNA in gel electrophoresis binding assays. Graphs show mean of three independently prepared samples + mean standard deviation of the distribution

All of the transfection‐optimized PBAE/DNA formulations used in later studies were characterized to assess the physicochemical properties of these leading nanoparticles. Transmission electron microscopy (TEM) images of the two leading nanoparticles in this study shows particles of approximately 100 nm diameter or slightly smaller (Figure 2a). Nanoparticle tracking analysis (NTA) supports the TEM findings, showing that all nanoparticle formulations had a number‐averaged mean hydrodynamic diameter between 100 and 150 nm (Figure 2b), with no apparent pattern relating to polymer type or polymer‐to‐DNA mass ratio. The small discrepancy in size from these two methods is likely due primarily to TEM measuring dried particles whereas NTA measures the hydrodynamic particle size in aqueous buffer.

The zeta potential of nanoparticles was found to be positive in all cases (Figure 2c), ranging from 9.0 ± 0.2 mV (PBAE 446) to 16 ± 1 or 16 ± 2 mV (PBAE 537 formulations). This was expected for nanoparticles composed of cationic polymers in excess of anionic DNA, and it is expected that the positive charge can help the particles to associate and be internalized by cells, which have relatively negative surfaces. Gel electrophoresis studies showed that all DNA was completely complexed with the cationic PBAEs (Figure 2d) in the formulations tested.

### 3.3 Biomaterial‐mediated cancer specificity

**Figure 3:**
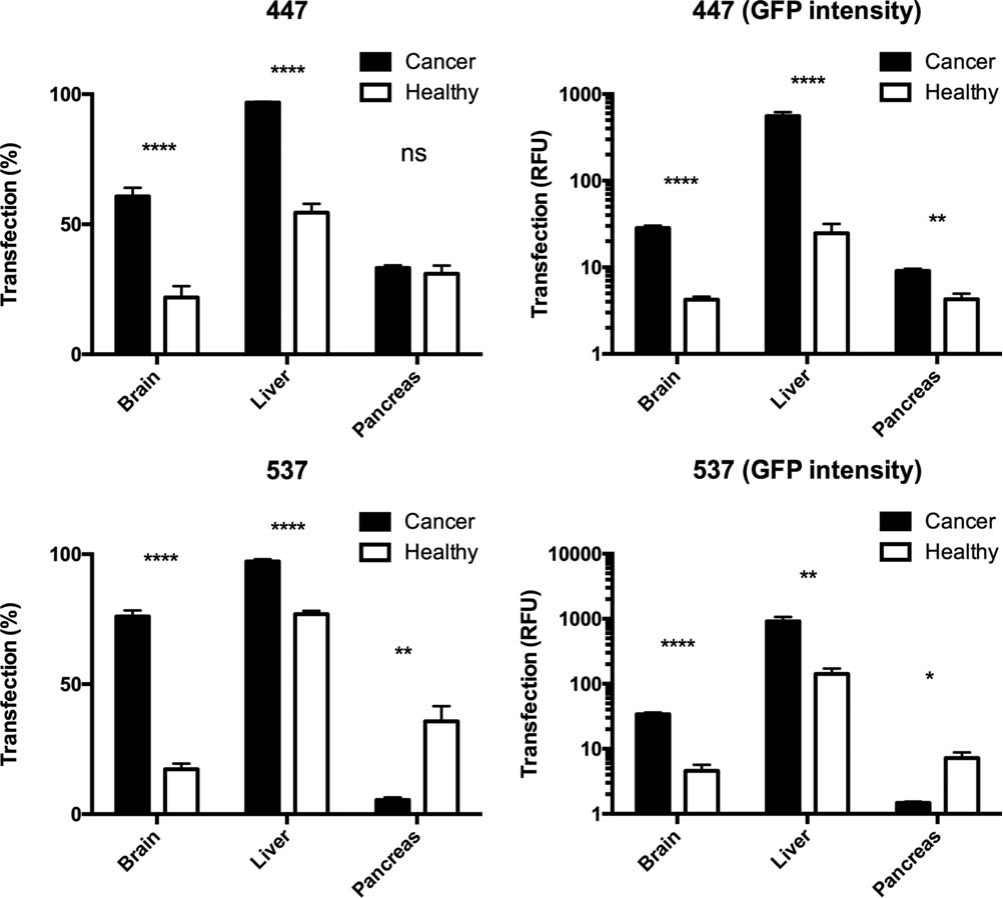
DNA‐loaded nanoparticles based on PBAEs 447 and 537 were used to transfect cancer and noncancer cells derived from human brain (JHGBM‐276 and F34), human pancreas (BxPC‐3 and hTERT‐HPNE) and rat liver (MCA‐RH7777 and BRL‐3A). Transfection efficacies are described as percent of cells positive for the transgene and as fluorescence intensity of the transfected GFP gene. The optimized nanoparticle formulations used for each pair of cell cultures are 447, 60 w/w and 537, 60 w/w (brain); 447, 75 w/w and 537, 75 w/w (liver); and 447, 50 w/w and 537, 75 w/w (pancreas)

Previous studies have shown that PBAE/DNA nanoparticles can transfect cancer cells significantly better than healthy cells, having been demonstrated in rat‐derived liver cells in vitro^17^ and in primary human brain cells both in vitro and in vivo.^19^ Further expanding on those observations, Figure 3 shows the cancer cell‐specificity of DNA‐loaded nanoparticles based on two different PBAEs (447 and 537). PBAE 447‐based particles showed statistically significantly better transfection in human brain and rat liver but not human pancreatic cancer cells compared with healthy cells of those same tissue types. In the case of the pancreatic cells, although transfection measured as percent of cells transfected was not significantly higher in cancer cells, the mean fluorescence intensity (normalized to autofluorescence in untreated cells) was significantly higher in pancreatic cancer cells for PBAE 447 particles (shown on a logarithmic scale in Figure 3).

Interestingly, while PBAE 537‐based nanoparticles were highly effective in brain and liver cancer cells, they showed little efficacy in pancreatic cancer cells while maintaining moderate efficacy in healthy pancreas cells. This may be related to a finding in previous work that 537‐based nanoparticles tended to be leading formulations in most brain cancer cell types but had exceptionally low efficacy in certain, particular patient‐derived cultures. As a result, this polymer may not be as universally useful in cancer applications as PBAE 447, but further studies of its properties may help to elucidate the mechanisms of cell‐specific efficacy across different PBAEs and cell types.

### 3.4 Cancer cell killing via delivery of TRAIL gene

Using the leading PBAE/DNA nanoparticle formulations found for each cell type, we delivered GFP‐TRAIL DNA to the human cancer cells used in this study to determine whether GFP‐TRAIL‐transfected cells showed higher cytotoxicity than GFP‐transfected cells. Because GFP is a relatively non‐toxic protein, and because the GFP‐TRAIL plasmid was originally created by cloning TRAIL into the same GFP plasmid, we expected that any differences in cell survival are due to TRAIL‐mediated apoptosis.

**Figure 4:**
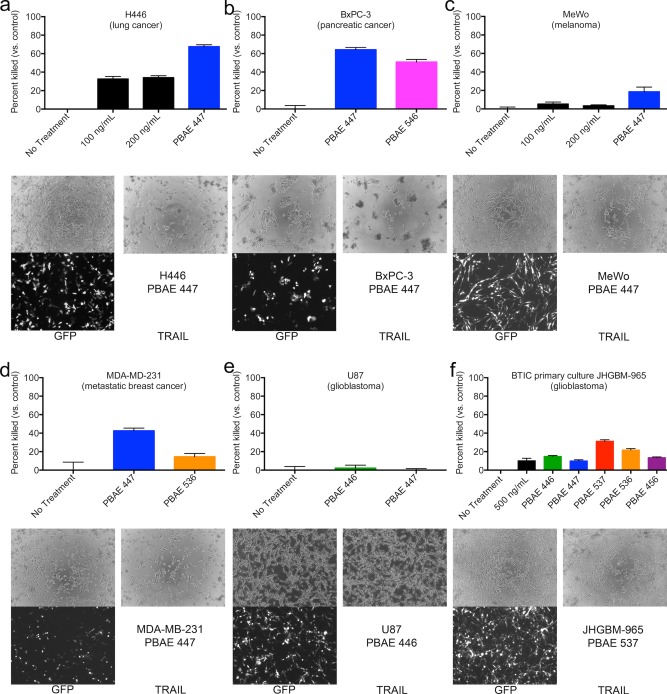
Y‐axes for all graphs show percent of cells killed by TRAIL activity assessed as relative metabolic activity of lung cancer (a), pancreatic cancer (b), melanoma (c), breast cancer (d), glioblastoma (e), and brain tumor initiating (f) cells transfected with GFP‐TRAIL, normalized to metabolic activity of cells transfected with GFP. For some of the cell lines that showed sensitivity to TRAIL transfection, soluble recombinant human TRAIL (rhTRAIL) was also tested. Microscopy images (10x magnification for all) are provided for one of the polymer conditions shown on the graphs, demonstrating with GFP that the cells were in fact transfected. Black bars represent cell death due to soluble rhTRAIL protein added to the media of untransfected cells

As shown in Figure 4, delivery of GFP‐TRAIL was very effective in killing cells in certain cancer cell lines evaluated, particularly small‐cell lung cancer line H446 and pancreatic cancer line BxPC‐3, with 68 ± 2% and 64 ± 3% cell death, respectively, when transfected with PBAE 447/TRAIL‐DNA (Table 3). Light microscopy showed high amounts of cell death in both of these groups compared with GFP‐transfected cells, in agreement with the quantitative measurements. The PBAE/TRAIL‐DNA treatment was significantly more effective (*p* < .0001) at killing H446 cells than treatment with recombinant human TRAIL (rhTRAIL), which showed no more than 34 ± 2% cell death when incubated with 200 ng/ml of soluble protein for 48 hr.

Other cell types, including MeWo melanoma cells, MDA breast cancer cells, and primary GBM cells, had very little or only moderate response to GFP‐TRAIL treatment (Figure 4), with only 13 ± 5%, 43 ± 3%, and 31 ± 1% cell death, respectively, while U87 human glioma cells showed no significant TRAIL‐mediated killing (2 ± 3% cell death). Importantly, as shown in Table 3 and qualitatively in Figure 4, all four of these cell lines had higher gene delivery efficacy, shown by transfection of GFP DNA, than TRAIL‐mediated killing. To ensure that this was not due simply to an unexpected effect of the biomaterial or nanoparticle, rhTRAIL was added to the media of JHGBM‐965 cells, one of the cell types refractory to GFP‐TRAIL transfection. As with H446 cells, even high concentrations of soluble rhTRAIL resulted in low TRAIL‐mediated killing, with 10 ± 3% cell death. Other primary cultures of human GBM lines were similarly evaluated and showed similarly low or even lower response to GFP‐TRAIL transfection, despite >50% transfection efficacy in some cultures (Table 3).

### 3.5 Expression of TRAIL and TRAIL‐binding receptors

**Figure 5:**
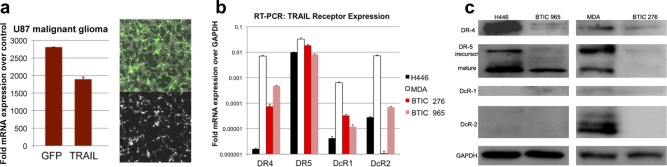
(a) PCR verification of TRAIL transcription in completely refractory cells that show high mRNA transcription, indicating successful transfection, but no TRAIL killing. (b) PCR assessment of mRNA transcript level of death receptor genes (DR4, DR5) and decoy receptor genes (DcR1, DcR2) in four cell types spanning the spectrum of TRAIL responsively was not well correlated with TRAIL sensitivity. (c) Western blot assessment of death and decoy receptor protein expression better correlated with TRAIL sensitivity

In order to better understand the reasons for low efficacy of TRAIL in certain cancer cell types, we measured the level of transfection using PCR to ensure that there was no unexpected inhibition of GFP‐TRAIL transcription. As seen in Figure 5A, even in U87 cells, which were completely refractory to GFP‐TRAIL treatment, the level of TRAIL mRNA in GFP‐TRAIL‐transfected cells was high and similar to the level of GFP mRNA in GFP‐transfected cells, confirming that the gene transfer step was in fact successful.

We also analyzed the expression of TRAIL‐binding receptors DR4 and DR5, which both lead to downstream apoptosis, and the decoy receptors DcR1 and DcR2, which both lack the intracellular death domain necessary to cause cell death. We examined four cell types spanning the spectrum of responsiveness to TRAIL, including H446 cells, on which TRAIL is highly effective; MDA and JHGBM‐965 cells, which show low to moderate response to TRAIL; and JHGBM‐276 cells, which showed no significant response to TRAIL. PCR was used to measure mRNA expression of the four receptors, and while the results are not directly comparable between cell types because of intrinsic differences in protein‐ and mRNA production levels, the expression of the four receptors within each cell line can be compared. Figure 5B shows that H446 cells had high mRNA expression of DR5, one of the death receptors, and low expression of both decoy receptors; MDA cells had high expression of mRNA for all four receptors, including death‐inducing and decoy receptors; and both BTIC GBM lines, surprisingly, had similar mRNA expression patterns to H446 cells, and in fact expressed more of the DR4 death receptor mRNA than H446.

Western blot analysis of these four proteins appeared to be more predictive of TRAIL sensitivity, as the protein expression levels corresponded well to transfection trends (Figure 5B). H446s showed very high DR4 protein expression, despite having low mRNA levels, as well as high DR5 and low DcR1 and DcR2 levels, in agreement with PCR. For MDA cells, moderate to high expression of all four receptors was found, matching PCR data as well as the moderate efficacy of TRAIL after transfection. Both of the GBM BTIC cell cultures showed lower decoy receptor protein expression than expected from PCR, but their low death receptor expression is consistent with the low cell death observed upon transfection with TRAIL‐encoding nanoparticles.

## 4 Discussion

In this work, we have shown that PBAE‐based nanoparticles can be effective for DNA delivery to all of the 10 cancer cell cultures that we evaluated, representing cells derived from six different tissue types. Moreover, our results demonstrate that PBAE/DNA nanoparticles can preferentially transfect cancer cells over healthy cells, resulting in either a higher fraction of cancer cells transfected or more protein produced by cancer cells compared with transfected healthy cells. This is an important and robust result that is promising for translation of these biodegradable nanoparticles for cancer gene therapy. The nanoparticles we used in this work all shared similar physicochemical properties, namely a hydrodynamic diameter between 100 and 150 nm and moderate but positive zeta potential, aspects that aid the particles' ability to interact with and be internalized by cells.^27^, ^28^ The mechanism of cancer specificity of these particles has not yet been elucidated. Previous work has shown that that the cancer specificity is not simply a result of differences in cellular doubling time or a result of differences to overall cellular uptake,^19^ and that different cellular uptake pathways lead to different transfection efficacy,^15^ which could affect the types of cells that are more affected by these nanoparticles.

We have also shown that delivery of GFP‐TRAIL DNA can be a useful therapeutic modality for certain cell lines and cancer types. In particular, lung and pancreatic cancer cells responded very well to transfection with TRAIL. Importantly, in the case of both of these cell types, the percent of cells expressing the transgene, measured via flow cytometry of GFP‐transfected cells, was only 32 ± 2% in H446 cells and 33 ± 1% in BxPC‐3 cells, much lower than the TRAIL‐mediated killing rates of 68 ± 2% and 64 ± 3%, respectively. This is most likely due to the known bystander effect of TRAIL expression: because TRAIL is cell surface‐bound ligand, it can affect neighboring cells, thereby causing apoptosis in more cells than were initially transfected.^26^ Conversely, the melanoma, breast cancer, and brain cancer cell lines used in this study showed lower TRAIL‐mediated killing rates than their transfection efficacy, indicating resistance to TRAIL.

Interestingly, both in cells highly responsive to TRAIL and in cells largely refractory to it, transfected cells were killed more effectively than cells exposed to relatively high levels of soluble rhTRAIL. This is likely due to the fact that GFP‐TRAIL, after transfection, is bound to cell surfaces. If more than one ligand is expressed on a single cell, this immobilization on cell membranes may improve binding avidity. This finding is in agreement with work by other groups showing that immobilized TRAIL was more effective in causing apoptosis than soluble TRAIL.^29^, ^30^

While cancer cells often overexpress death receptors DR4 and DR5, healthy cells often overexpress the decoy receptors DcR1 and DcR2 as a mechanism to prevent undesired apoptosis.^31^, ^32^ However, studies have shown that one mechanism of resistance to TRAIL is to decrease death domain activation by either downregulating or mutating DR4/DR5 or upregulating the protective DcR1/DcR2 receptors.^33^ Western blot analysis showed that H446 cells indeed did express high levels of both death receptors and no detectable levels of the decoys, explaining their responsiveness to TRAIL treatment. MDA cells may be partially resistant due to the expression of decoy receptors, although the presence of death receptors in MDA cells does still allow for some efficacy of TRAIL treatment. Critically, the GBM cells both showed low or undetectable levels of death receptor protein expression, although JHGBM‐965 cells had some DR5 expression. This finding is consistent with the low efficacy of TRAIL on the GBM cells in general, as well as the slightly higher efficacy in JHGBM‐965 cells compared to JHGBM‐276 and other GBM cells tested. Although mRNA expression levels from PCR did not always agree with protein electrophoresis data, this was consistent with other groups' results, which showed imperfect correlation between mRNA expression of these four TRAIL receptors and even, in some cases, what appears to be contradictory data.^34^, ^35^, ^36^ Future studies could use RNAi‐mediated knockdown of death and/or decoy receptors to further elucidate the mechanism of resistance to TRAIL in these cell types. A combination treatment of siRNA against TRAIL decoy receptors combined with DNA‐encoding TRAIL could also be an interesting potential therapeutic modality.

We have demonstrated that non‐viral polymeric delivery of TRAIL DNA can be a promising therapeutic strategy for certain cancer types. In addition, our group has demonstrated that PBAEs can effectively deliver DNA to tumors following local administration in vivo.^19^, ^20^ Moreover, the ability to deliver DNA, such as TRAIL, with biomaterial‐mediated cancer cell specificity suggests this system's potential utility in specifically transfecting cancer cells with this therapeutic gene even in an in vivo environment. For a systemic in vivo administration, these PBAE nanoparticles may need to be surface coated to improve their biodistribution and their tissue targeting.^37^, ^38^ Alternatively, they could be utilized to transfect cells ex vivo that have the capacity to migrate to tumors in vivo as part of a genetically engineered cellular therapy.^39^ Although there are various mechanisms of resistance, including upregulation of decoy receptors, as in the case of breast cancer cells, or downregulation of death receptors, as in the GBM cells, the lung and pancreatic cancer cell lines tested were killed very effectively by TRAIL. Protein expression analysis may be a reliable method of assessing whether a cell sample will respond to TRAIL treatment. Non‐viral PBAE/TRAIL nanoparticles have the potential to be therapeutic modalities for the treatment of lung cancer and pancreatic cancer.

## Supporting information

Supporting Information

## References

[1] Wu W , Sun M , Zou GM , Chen J. MicroRNA and cancer: current status and prospective. Int J Cancer. 2007;120(5):953–960. 17163415 10.1002/ijc.22454

[2] Yadav S , van Vlerken LE , Little SR , Amiji MM. Evaluations of combination MDR‐1 gene silencing and paclitaxel administration in biodegradable polymeric nanoparticle formulations to overcome multidrug resistance in cancer cells. Cancer Chemother Pharmacol. 2009;63(4):711–722. 18618115 10.1007/s00280-008-0790-y

[3] Nguyen DN , Green JJ , Chan JM , Langer R , Anderson DG. Polymeric materials for gene delivery and DNA vaccination. Adv Mater. 2009;21(8):847–867. 28413262 10.1002/adma.200801478 PMC5391878

[4] Pringle IA , Hyde SC , Gill DR. Non‐viral vectors in cystic fibrosis gene therapy: recent developments and future prospects. Expert Opin Biol Ther. 2009;9(8):991–1003. 19545217 10.1517/14712590903055029

[5] Ptasznik A , Nakata Y , Kalota A , Emerson SG , Gewirtz AM. Short interfering RNA (siRNA) targeting the Lyn kinase induces apoptosis in primary, and drug‐resistant, BCR‐ABL1(+) leukemia cells. Nat Med. 2004;10(11):1187–1189. 15502840 10.1038/nm1127

[6] Kim SH , Jeong JH , Lee SH , Kim SW , Park TG. Local and systemic delivery of VEGF siRNA using polyelectrolyte complex micelles for effective treatment of cancer. J Control Release. 2008;129(2):107–116. 18486981 10.1016/j.jconrel.2008.03.008

[7] Howard KA , Paludan SR , Behlke MA , Besenbacher F , Deleuran B , Kjems J. Chitosan/siRNA nanoparticle‐mediated TNF‐alpha knockdown in peritoneal macrophages for anti‐inflammatory treatment in a murine arthritis model. Mol Ther. 2009;17(1):162–168. 18827803 10.1038/mt.2008.220 PMC2834976

[8] Karvinen H , Yla‐Herttuala S. New aspects in vascular gene therapy. Curr Opin Pharmacol. 2010;10(2):208–211. 20163988 10.1016/j.coph.2010.01.004

[9] Tobias A , Ahmed A , Moon KS , Lesniak MS. The art of gene therapy for glioma: a review of the challenging road to the bedside. J Neurol Neurosurg Psychiatr. 2013;84(2):213–222. 22993449 10.1136/jnnp-2012-302946 PMC3543505

[10] Thomas CE , Ehrhardt A , Kay MA. Progress and problems with the use of viral vectors for gene therapy. Nat Rev Genet. 2003;4(5):346–358. 12728277 10.1038/nrg1066

[11] Pack DW , Hoffman AS , Pun S , Stayton PS. Design and development of polymers for gene delivery. Nat Rev Drug Discov. 2005;4(7):581–593. 16052241 10.1038/nrd1775

[12] Viola JR , El‐Andaloussi S , Oprea II , Smith CIE. Non‐viral nanovectors for gene delivery: factors that govern successful therapeutics. Expert Opin Drug Deliv. 2010;7(6):721–735. 20367531 10.1517/17425241003716810

[13] Sunshine JC , Bishop CJ , Green JJ. Advances in polymeric and inorganic vectors for nonviral nucleic acid delivery. Ther Deliv. 2011;2(4):493–521. 22826857 10.4155/tde.11.14 PMC4000586

[14] Bishop CJ , Ketola TM , Tzeng SY , et al. The effect and role of carbon atoms in poly(beta‐amino ester)s for DNA binding and gene delivery. J Am Chem Soc. 2013;135(18):6951–6957. 23570657 10.1021/ja4002376 PMC3838887

[15] Kim J , Sunshine JC , Green JJ. Differential polymer structure tunes mechanism of cellular uptake and transfection routes of poly(beta‐amino ester) polyplexes in human breast cancer cells. Bioconjug Chem. 2014;25(1):43–51. 24320687 10.1021/bc4002322 PMC4016154

[16] Tzeng SY , Guerrero‐Cazares H , Martinez EE , Sunshine JC , Quinones‐Hinojosa A , Green JJ. Non‐viral gene delivery nanoparticles based on poly(beta‐amino esters) for treatment of glioblastoma. Biomaterials. 2011;32(23):5402–5410. 21536325 10.1016/j.biomaterials.2011.04.016 PMC3118545

[17] Tzeng SY , Green JJ. Subtle changes to polymer structure and degradation mechanism enable highly effective nanoparticles for siRNA and DNA delivery to human brain cancer. Adv Healthc Mater. 2013;2(3):468–480. 23184674 10.1002/adhm.201200257 PMC3838886

[18] Sunshine JC , Sunshine SB , Bhutto I , Handa JT , Green JJ. Poly (beta‐amino ester)‐nanoparticle mediated transfection of retinal pigment epithelial cells in vitro and in vivo. PloS one. 2012;7(5):e37543. 22629417 10.1371/journal.pone.0037543 PMC3357345

[19] Guerrero‐Cazares H , Tzeng SY , Young NP , Abutaleb AO , Quinones‐Hinojosa A , Green JJ. Biodegradable polymeric nanoparticles show high efficacy and specificity at DNA delivery to human glioblastoma in vitro and in vivo. ACS Nano. 2014;8(5):5141–5153. 24766032 10.1021/nn501197v PMC4046784

[20] Kamat CD , Shmueli RB , Connis N , Rudin CM , Green JJ , Hann CL. Poly(beta‐amino ester) nanoparticle delivery of TP53 has activity against small cell lung cancer in vitro and in vivo. Mol Cancer Ther. 2013;12(4):405–415. 23364678 10.1158/1535-7163.MCT-12-0956 PMC3624031

[21] Mangraviti A , Tzeng SY , Kozielski KL , et al. Polymeric nanoparticles for nonviral gene therapy extend brain tumor survival in vivo. ACS Nano. 2015;9(2):1236–1249. 25643235 10.1021/nn504905q PMC4342728

[22] Shmueli RB , Sunshine JC , Xu Z , Duh EJ , Green JJ. Gene delivery nanoparticles specific for human microvasculature and macrovasculature. Nanomedicine. 2012;8(7):1200–1207. 22306159 10.1016/j.nano.2012.01.006 PMC3350835

[23] Sunshine J , Green JJ , Mahon KP , et al. Small molecule end group of linear polymer determines cell‐type gene delivery efficacy. Adv Mater. 2009;21(48):4947–4951. 25165411 10.1002/adma.200901718 PMC4143259

[24] Kim J , Wilson DR , Zamboni CG , Green JJ. Targeted polymeric nanoparticles for cancer gene therapy. J Drug Target. 2015;23(7–8):627–641. 26061296 10.3109/1061186X.2015.1048519 PMC4696040

[25] Dimberg LY , Anderson CK , Camidge R , Behbakht K , Thorburn A , Ford HL. On the TRAIL to successful cancer therapy[quest] Predicting and counteracting resistance against TRAIL‐based therapeutics. Oncogene. 2013;32(11):1341–1350. 22580613 10.1038/onc.2012.164 PMC4502956

[26] Kagawa S , He C , Gu J , et al. Antitumor activity and bystander effects of the tumor necrosis factor‐related apoptosis‐inducing ligand (TRAIL) gene. Cancer Res. 2001;61(8):3330–3338. 11309289

[27] Win KY , Feng SS. Effects of particle size and surface coating on cellular uptake of polymeric nanoparticles for oral delivery of anticancer drugs. Biomaterials. 2005;26(15):2713–2722. 15585275 10.1016/j.biomaterials.2004.07.050

[28] Jiang W , Kim BY , Rutka JT , Chan WC. Nanoparticle‐mediated cellular response is size‐dependent. Nat Nanotechnol. 2008;3(3):145–150. 18654486 10.1038/nnano.2008.30

[29] Balyasnikova IV , Ferguson SD , Han Y , Liu FF , Lesniak MS. Therapeutic effect of neural stem cells expressing TRAIL and bortezomib in mice with glioma xenografts. Cancer Lett. 2011;310(2):148–159. 21802840 10.1016/j.canlet.2011.06.029 PMC3159776

[30] Voelkel‐Johnson C , King DL , Norris JS. Resistance of prostate cancer cells to soluble TNF‐related apoptosis‐inducing ligand (TRAIL/Apo2L) can be overcome by doxorubicin or adenoviral delivery of full‐length TRAIL. Cancer Gene Ther. 2002;9(2):164–172. 11857034 10.1038/sj.cgt.7700420

[31] Walczak H , Miller RE , Ariail K , et al. Tumoricidal activity of tumor necrosis factor related apoptosis‐inducing ligand in vivo. Nat Med. 1999;5(2):157–163. 9930862 10.1038/5517

[32] Hao CH , Beguinot F , Condorelli C , et al. Induction and intracellular regulation of tumor necrosis factor‐related apoptosis‐inducing ligand (TRAIL) mediated apotosis in human malignant glioma cells. Cancer Res. 2001;61(3):1162–1170. 11221847

[33] Zhang LD , Fang BL. Mechanisms of resistance to TRAIL‐induced apoptosis in cancer. Cancer Gene Ther. 2005;12(3):228–237. 15550937 10.1038/sj.cgt.7700792

[34] Griffith TS , Chin WA , Jackson GC , Lynch DH , Kubin MZ. Intracellular regulation of TRAIL‐induced apoptosis in human melanoma cells. J Immunol. 1998;161(6):2833–2840. 9743343

[35] Eggert A , Grotzer MA , Zuzak TJ , et al. Resistance to tumor necrosis factor‐related apoptosis‐inducing ligand (TRAIL)‐induced apoptosis in neuroblastoma cells correlates with a loss of caspase‐8 expression. Cancer Res. 2001;61(4):1314–1319. 11245427

[36] Jin ZY , McDonald ER , Dicker DT , El‐Deiry WS. Deficient tumor necrosis factor‐related apoptosis‐inducing ligand (TRAIL) death receptor transport to the cell surface in human colon cancer cells selected for resistance to TRAIL‐induced apoptosis. J Biol Chem. 2004;279(34):35829–35839. 15155747 10.1074/jbc.M405538200

[37] Harris TJ , Green JJ , Fung PW , Langer R , Anderson DG , Bhatia SN. Tissue‐specific gene delivery via nanoparticle coating. Biomaterials. 2010;31(5):998–1006. 19850333 10.1016/j.biomaterials.2009.10.012 PMC2796451

[38] Kim J , Kang Y , Tzeng SY , Green JJ. Synthesis and application of poly(ethylene glycol)‐co‐poly(beta‐amino ester) copolymers for small cell lung cancer gene therapy. Acta Biomater. 2016. 10.1016/j.actbio.2016.05.040 PMC6061916 27262740

[39] Mangraviti A , Tzeng SY , Gullotti D , et al. Non‐virally engineered human adipose mesenchymal stem cells produce BMP4, target brain tumors, and extend survival. Biomaterials. 2016;100:53–66. 27240162 10.1016/j.biomaterials.2016.05.025 PMC4902753

