# Supplementary material for: Polymeric nanoparticle‐based delivery of TRAIL DNA for cancer‐specific killing
Source: Bioeng Transl Med. 2016 Aug 19;1(2):149–59. doi: 10.1002/btm2.10019 (PMC5365091; doi:10.1002/btm2.10019)
Supplement: Supplementary file 1 — Supporting Information [file BTM2-1-149-s001.pdf]

Supplemental Information

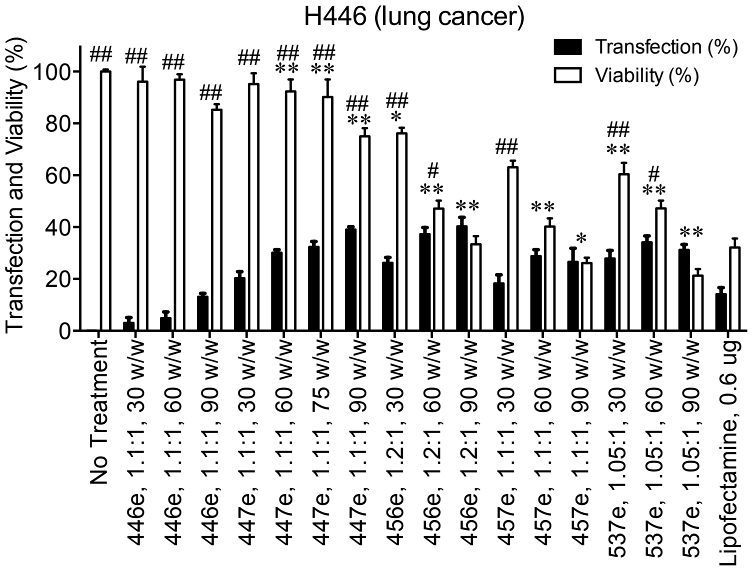

Supplemental Figure 1. H446 lung cancer cells were transfected with PBAEs at a DNA dose of 0.6 μg/well. Transfection efficacy and viability of the cells were analyzed to find the optimal nanoparticle formulation. Lipofectamine™ 2000 (Lipo) was tested at the same DNA dosage as PBAEs (0.6 μg). One-way ANOVA showed that some PBAE-based formulations were statistically significantly better in transfection (*) or viability (#) than Lipo tested at the same dosage. * and #: $p<0.05$; ** and ##: $p<0.01$.

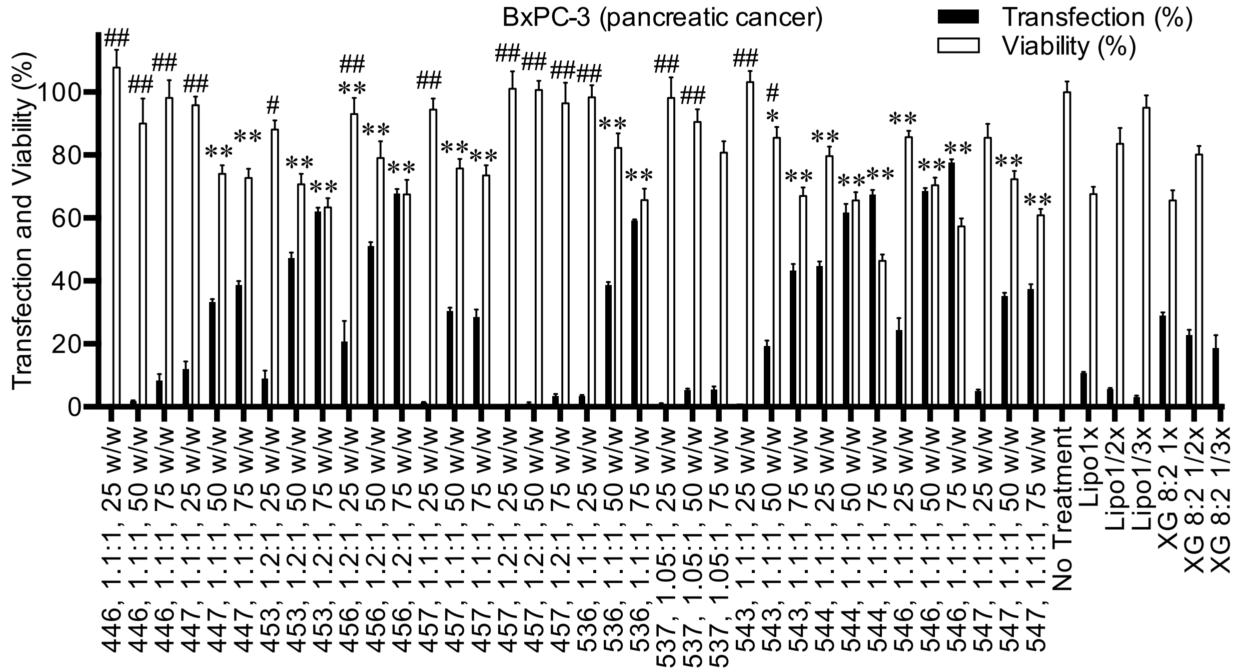

Supplemental Figure 2. BxPC-3 pancreatic cancer cells were transfected with PBAEs at a DNA dose of 0.6 μg/well. Transfection efficacy and viability of the cells were analyzed to find the optimal nanoparticle formulation. Lipofectamine™ 2000 (Lipo) was tested at the same DNA dosage as PBAEs (1x) and at lower doses, using the manufacturer's recommended formulation. XtremeGENE HP DNA (XG) was tested at a 8:2 ratio of reagent to DNA at three different doses. One-way ANOVA showed that some PBAE-based formulations were statistically significantly better in transfection (*) or viability (#) than Lipo tested at the same dosage. * and ##: $p<0.05$; ** and #: $p<0.01$.

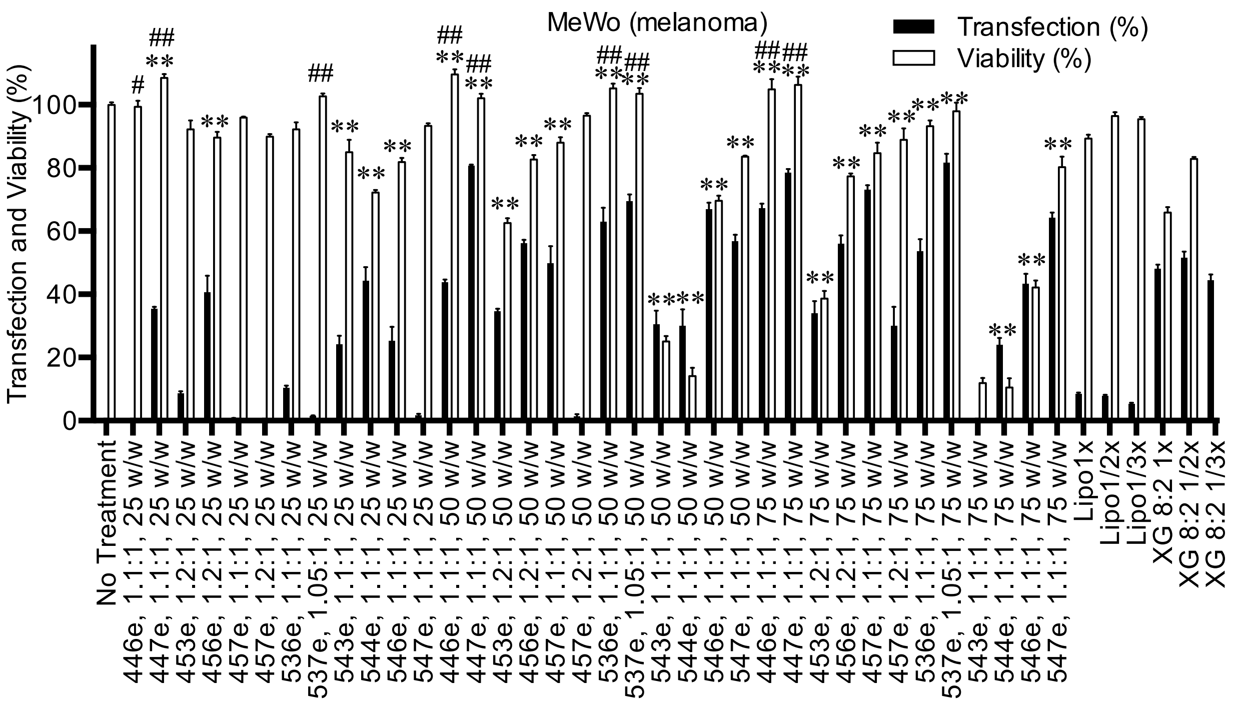

Supplemental Figure 3. MeWo melanoma cells were transfected with PBAEs at a DNA dose of 0.6 μg/well. Transfection efficacy and viability of the cells were analyzed to find the optimal nanoparticle formulation. Lipofectamine™ 2000 (Lipo) was tested at the same DNA dosage as PBAEs (0.6 μg) and at lower doses, using the manufacturer's recommended formulation. XtremeGENE HP DNA (XG) was tested at a 8:2 ratio of reagent to DNA at three different doses. One-way ANOVA showed that some PBAE-based formulations were statistically significantly better in transfection (*) or viability (#) than Lipo tested at the same dosage. * and #: $p<0.05$; ** and ##: $p<0.01$.

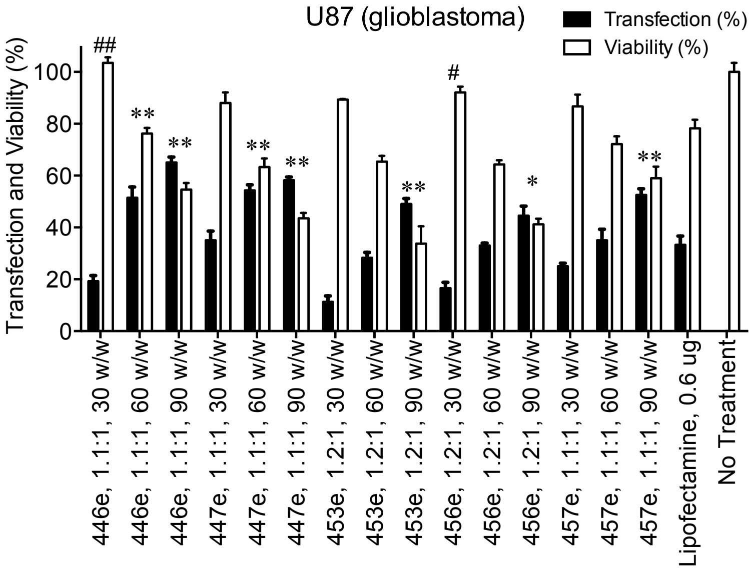

Supplemental Figure 4. U87 glioblastoma cells were transfected with PBAEs at a DNA dose of 0.6 μg/well. Transfection efficacy and viability of the cells were analyzed to find the optimal nanoparticle formulation. Lipofectamine™ 2000 (Lipo) was tested at the same DNA dosage as PBAEs (0.6 μg). One-way ANOVA showed that some PBAE-based formulations were statistically significantly better in transfection (*) or viability (#) than Lipo tested at the same dosage. * and #: $p<0.05$; ** and ##: $p<0.01$.
